# Conditionally Rare Taxa Contribute but Do Not Account for Changes in Soil Prokaryotic Community Structure

**DOI:** 10.3389/fmicb.2018.00809

**Published:** 2018-04-27

**Authors:** Rachel Kaminsky, Sergio E. Morales

**Affiliations:** Department of Microbiology and Immunology, University of Otago, Dunedin, New Zealand

**Keywords:** conditionally rare taxa, rare biosphere, 16S rRNA, pH, land use, soil classification, seasonal dynamics

## Abstract

The rare biosphere is predicted to aid in maintaining functional redundancy as well as contributing to community turnover across many environments. Recent developments have partially confirmed these hypotheses, while also giving new insights into dormancy and activity among rare communities. However, less attention has been paid to the rare biosphere in soils. This study provides insight into the rare biosphere’s contribution to soil microbial diversity through the study of 781 soil samples representing 24 edaphically diverse sites. Results show that Bray–Curtis dissimilarity for time-sensitive conditionally rare taxa (CRT) does not correlate with whole community dissimilarity, while dissimilarity for space-sensitive CRT only weakly correlate with whole community dissimilarity. This adds to current understanding of spatiotemporal filtering of rare taxa, showing that CRT do not account for community variance across tested soils, but are under the same selective pressure as the whole community.

## Introduction

Rare species are pervasive in microbial consortia, comprising the majority of microbial species (Curtis et al., 2002). Known as the “rare biosphere,” these members are being increasingly recognized for their importance to maintaining alpha diversity in microbial communities, as well as key ecosystem functions (Pedrós-Alió, 2007; Lynch and Neufeld, 2015). There are several potential causes of rarity including transience (where organisms inhabit a niche stochastically, as with dispersal, but aren’t adapted enough to flourish), competition for resources, niche breadth and predation of abundant taxa (Jousset et al., 2017). It has been suggested that the rare biosphere is a dormant “seed bank” wherein members become abundant when formerly abundant taxa are vulnerable (Lennon and Jones, 2011). As such, rare members are thought to contribute to both functional redundancy and community turnover across many environments (Pedrós-Alió, 2007).

Despite the prominence of the seed bank theory, rare microbes can also be active (Campbell et al., 2011; Hugoni et al., 2013; Kurm et al., 2017), signifying several potential ecological roles for the rare biosphere. Jousset et al. (2017) proposed three possible functions for rare members. These include: biochemical processes such as nutrient cycling, community assembly, including resistance to disturbance or invasion and driving the functions of host-associated microbiomes, particularly host immunity. However, many studies opt to only investigate the abundant portion of the microbial biosphere (Jousset et al., 2017). Consequently, the details of these functions remain unresolved raising many questions about the contribution and ecological importance of rare members.

Recent work identified conditionally rare taxa (CRT), microbes that are rare at certain points in time and space and “bloom” to abundance at other points, as major contributors to community dynamics in several environments (Shade et al., 2014). This has given insight into the mechanisms that underlie the rare biosphere, confirming that some rare microbes can become abundant and are potentially responsible for changes in community structure and composition over space and time. In soils, studies have found that rare microbes bloom in response to disturbances (Aanderud et al., 2015; Fuentes et al., 2016). Despite these important findings, the literature is still developing with regard to the soil rare biosphere. Given the importance of soil microbial communities to mediating ecosystem processes, and the significant impact that land management has on soil ecosystems (e.g., Lauber et al., 2013; Kaminsky et al., 2017), understanding the contribution of rare soil microbes is of great importance. Here, we investigate CRT specifically because of the “bloom” characteristic. Because these taxa become abundant, concerns surrounding sequencing error given the low density of rare taxa are mitigated.

This study aims to investigate the contribution of conditionally rare prokaryotes to community variance over space and time using agricultural soils as a model system. We sampled 781 soils from 24 sites under three agricultural practices (dairy/high intensity, sheep + beef/medium intensity and high country/low intensity) in New Zealand to observe community differences between practices of differing intensities. Sampling occurred during three time points over a year (May 2014, November 2014, and May 2015) to capture the most divergent seasonal stages (summer to winter and return to summer) to assess community changes over space and time. Using 16S profiles, we tested two hypotheses: (H_1_) CRT contribute disproportionately to changes in whole community variance and (H_2_) recruitment from the rare biosphere is linked to spatiotemporal filters.

## Materials and Methods

### Soil Sampling

Soil samples were collected from 24 sites representing three agricultural practices (dairy-high intensity, sheep + beef-medium intensity and high country-low intensity) in four geographic regions (North Canterbury, South Canterbury, Otago, and Southland). Samples were taken in May 2014, November 2014, and May 2015 to represent the beginning and end of the growing season in New Zealand. We sampled the same month in both 2014 and 2015 to observe the ability of prokaryotic communities to withstand seasonal fluctuation. Sites consisted of twelve replicate plots (1 m^2^ each) within a fence. Each sample is a composite of four cores (7.5 cm depth and 2.5 cm diameter) that were taken 0.4 m apart diagonally across each plot. There are a total of 864 samples in the study (24 sites × 12 plots × 3 time points). A table describing the sampling scheme is provided in Supplementary Table S1. Samples were stored at -80°C prior to DNA extraction. Data from the May 2014 time point were previously published in a separate study (Kaminsky et al., 2017). This paper found that pH, land use, and soil order account for the majority of community variance, hence these are the variables we chose to evaluate here. A full account of chemical data is provided in Supplementary Table S2. Additional cores were taken from each site for chemical analyses. Soils were also classified at the soil order and subgroup level using the New Zealand Soil Classification (Hewitt, 2010). Soil classification is of interest in studies of managed soils as it encompasses several inherent soil properties such as particle size, parent material and major chemical traits. As such, it may be useful in developing sustainable management practices.

### DNA Extraction

Genomic DNA was extracted from 0.25 g of soil using the Mo Bio PowerSoil-htp 96-well soil DNA isolation kit (Carlsbad, CA, United States) according to the manufacturer’s instructions, but using a Geno/Grinder homogenizer (SPEX SamplePrep, LLC, Metuchen, NJ, United States) during the lysing step for two rounds of 15 s at 1750 strokes/minute. DNA concentration was quantified using a NanoDrop 1000 Spectrophotometer (Thermo Scientific, Wilmington, DE, United States).

The V4 region of the 16S rRNA gene was amplified using the universal primer pair 515F (5′-NNNNNNNNGTGTGCCAGCMGCCGCGGTAA-3′) and 806 R (5′-GGACTACHVGGGTWTCTAAT-3′) following the Earth Microbiome Project barcoded conditions (Caporaso et al., 2012). Each sample was given a barcode sequence on the 5′ end of the forward primer for multiplexed sequencing and loaded onto a single Illumina MiSeq 2 × 151 bp run (Illumina, Inc., San Diego, CA, United States). Sequences were deposited at the Sequence Read Archive (NCBI) with the accession numbers: 5902515–5902586 under the BioProject ID: PRJNA348131 and 5801200–5801546, 5803240–5803606 under the BioProject ID: PRJNA391831.

### Sequencing Processing

Data from all time points were collated and processed using an open reference protocol in QIIME version 1.9.1 (Caporaso et al., 2010) and release 123 of the SILVA database (Quast et al., 2013). The resulting OTU table was subsampled to 12,000 sequences per sample. 83 samples had fewer than 12,000 sequences and were removed at this step. The resulting OTU table contained data from 24 farms across three time points and three land uses. Each site has between 6 and 12 replicates per time point, for a total of 781 samples.

### Statistical Analyses

All analyses were performed in R version 3.3.2 (R Development Core Team, 2008; RStudio, 2016). The data were imported into R and divided by site using phyloseq (McMurdie and Holmes, 2013). All plots were made using ggplot2 (Wickham, 2009). Mantel (999 permutations) and ANOSIM tests were performed in vegan (Oksanen et al., 2017). Correlations between individual taxa and spatiotemporal factors were determined using ALDEx2 (Fernandes et al., 2013) by the Kruskal–Wallis test (soil order, land use, and time point) and Spearman’s rank correlation (pH) on log-ratio transformed data with 128 Monte-Carlo permutations. All taxa that correlated with spatial factors were plotted, while only the most significant taxon for each site was plotted for time. All taxa that vary significantly with time are reported in Supplementary Table S5.

### Isolating CRT

To isolate CRT that only change across time, the full OTU table was divided by site, creating 24 separate OTU tables. Reads from site replicates were averaged under each time point to control for intra-site spatial effects (Supplementary Figure S1). Rank abundance curves of the collated OTU tables were plotted (Supplementary Figure S2). A relative abundance table excluding singletons was made using makeRFtable.f. CRT were detected using SimpleRareToPrev.f with an abundance threshold of 0.0001 and a *b-*value of 0.9 (Shade et al., 2014). The site-level OTU tables containing all replicates were then culled to only include CRT. To detect space-sensitive CRT, the same procedure was carried out again on the full OTU table without dividing by individual sites (Supplementary Figures S1, S3). Reads from samples across time were averaged (ex. reads from sample 1 were averaged across the three time points) to determine CRT that only change across space. We also performed analyses on CRT that change across both space and time using a data set that was not filtered by space- or time-responsive CRT. Results did not differ from those presented here, so we only include analyses for space- and time-responsive CRT. We felt that this analysis was more precise as we could explore spatial and temporal dynamics separately. All R scripts used here are available on GitHub: https://github.com/rachelkaminsky2691/RK_CRT.

## Results and Discussion

**FIGURE 1:**
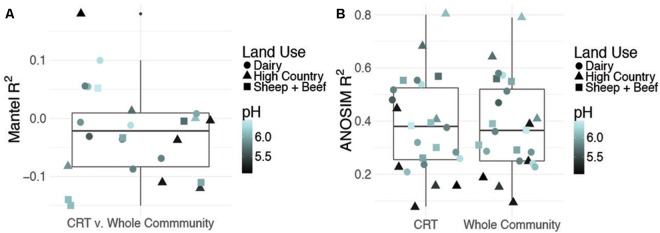
Contribution of conditionally rare taxa (CRT) to community variance. Summary of Mantel correlations between Bray–Curtis distances for site-level time responsive CRT communities and whole communities for each site **(A)** and summary of ANOSIM results for correlation between time and either site-level CRT or site-level whole community changes **(B)**. pH and land use are shown to discount confounding effects by a dominant soil driver.

Time responsive CRT constituted 4–6% of OTUs in each site while space responsive CRT represented 1% of the total community (*b-*value, >0.9, relative abundance >0.01%, Supplementary Figures S1–S3). To assess the contribution of CRT to whole community structure, we constructed three Bray–Curtis distance matrices from OTU tables for each site: (1) only OTUs identified as CRT, (2) whole communities including CRT, and (3) whole communities excluding CRT. Mantel tests between distance matrices for CRT and whole communities including CRT are insignificant, indicating that CRT do not drive prokaryotic community changes over time (**Figure 1A**). Further, the correlation between space responsive CRT and the whole community including CRT is significant, but weak (*R*^2^ = 0.02, *P* = 0.002). This shows that CRT account for more community variance over space than time, but not to the overwhelming degree that would be expected. Mantel tests between communities excluding CRT and whole communities including CRT have *R*^2-^values close to one (Supplementary Table S3). Overall, these results indicate that CRT do not drive community variation over space or time, which does not align with previous findings that postulate a larger role for rare microbes in community dynamics.

Previous work includes the Shade et al. (2014) CRT study that used moving window analysis, which is not employed here. This technique partitions the data into “windows” based on the number of time points and calculates the OTU richness for each window (White et al., 2006). CRT are then determined within the windows. This accounts for more variation in the data over time, allowing for consistent calculation of CRT across the study (Shade et al., 2014). Additionally, in our study, the time series consists of three time points, whereas Shade et al. (2014) employed datasets with many more time points sampled over a shorter time frame. The time series in this study samples seasonal extremes, and should capture the preponderance of variance expected for the time frame of interest, but having only three time points hinders our ability to predict consistent patterns of temporal CRT blooms, as we can’t account for taxa that may bloom on shorter time scales, as in moving window analysis. Furthermore, other studies of CRT used measurements of activity (Aanderud et al., 2015; Fuentes et al., 2016), which were not employed here. This would give more insight into the functional capacity of CRT, and how this relates to their contribution to overall community variance. Results regarding space-sensitive CRT are statistically well supported by a thorough sampling of a broad geographic range, indicating that CRT do not account for spatial differences.

Given the results at hand, it is possible that soil CRT have a limited role in soil functions, remaining mostly dormant and only blooming to abundance in extreme cases, as it is estimated that a substantial portion of microbes are inactive (Lennon and Jones, 2011). Alternatively, CRT might be K-selected, investing in few members that survive for longer, exhibiting a life strategy that is not reflected in whole community dynamics. This may be favorable given the heterogeneity of soil, wherein CRT may not overtake dominant taxa, but perform key functions that are costly for those taxa. For example, *Desulfosporosinus* is estimated to perform the majority of sulfate-reduction in peatlands despite its relatively minor contribution to community variance (Pester et al., 2010). Rare microbes cultivated from soils have also been found to play important roles in nitrogen fixation (Shade et al., 2012), providing further evidence for the potential disconnect between abundance and functional capacity.

**FIGURE 2:**
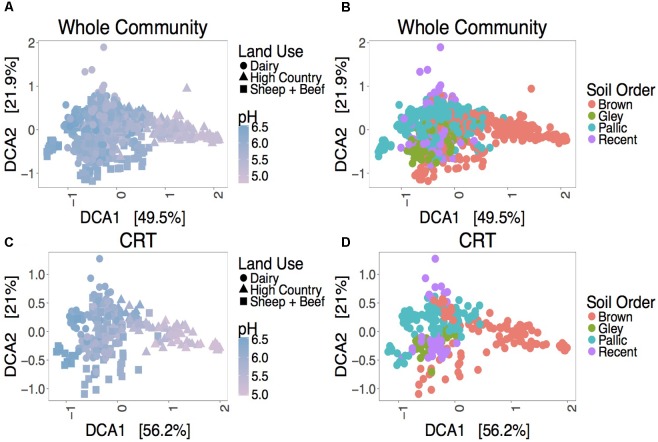
Ordination plots showing relationships between the whole community **(A,B)**, space responsive CRT **(C,D)** and spatial factors. DCA ordinations are based on Bray–Curtis distance matrices.

Despite a minor contribution to whole community variance, CRT community structure is linked to spatiotemporal factors. ANOSIM tests revealed significant correlations for CRT and whole communities at individual sites with time (**Figure 1B** and Supplementary Figures S4, S5). Mantel tests between space responsive CRT, the whole community and pH were significant, and ANOSIM tests with land use and soil order were also significant (**Figure 2**, Supplementary Figure S6, and Supplementary Table S4). These results agree with previous studies which found that soil prokaryotic communities exhibit temporal patterns (Lauber et al., 2013), are sensitive to pH change (Lauber et al., 2009), land use change (Steenwerth et al., 2002), and soil type (Kaminsky et al., 2017). As was mentioned previously, the analysis method employed here is impartial, detecting taxa that “bloom” across time or space without associating the bloom to particular niche factors. Given that CRT community variance is associated with pH, land use, and soil order, it can be posited that soil CRT follow the same assembly rules as abundant taxa. This indicates that CRT contribute to community changes, despite not being overwhelmingly represented in these dynamics.

**FIGURE 3:**
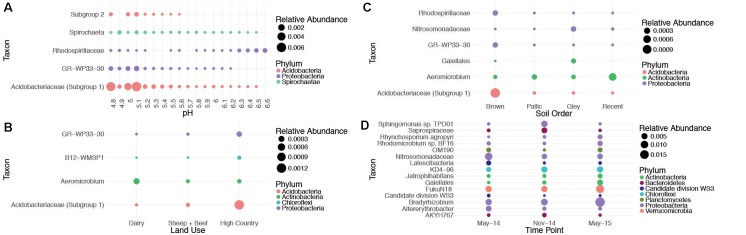
Relationships between individual CRT and key spatiotemporal factors. OTUs identified as CRT and significantly correlated to changes in pH **(A)**, land use **(B)**, soil order **(C)**, and time point **(D)**. The OTU from each site that varied most significantly with time was plotted. A full list of OTUs correlated with time is reported in Supplementary Table S5. Taxa were chosen based on a Kruskal–Wallis Benjamini-Hochberg adjusted *P* < 0.05 (land use, soil order, and time), and a Spearman’s Benjamini-Hochberg adjusted *P* < 0.05 for pH. Taxa are presented at the lowest classification level available, and colored at the phylum level.

Although CRT communities exhibit broad relationships with spatiotemporal factors, our results revealed that only 8% of time responsive CRT and 0.005% of space responsive CRT were found to be correlated to measured spatiotemporal factors (**Figure 3**). Key taxa are represented; for example, *Acidobacteria* is widely known to be sensitive to pH, and is reflected as such here, where it is rare in high pH soils and abundant in low pH soils. *Saprospiraceae* vary seasonally, which is consistent with previous findings (Schauer et al., 2006). These results may support the hypothesis that certain rare members have major functional roles in soils, but are not well represented in overall community variance. Further it is possible that ecological filters not accounted for here, such as neutral processes or unmeasured niche factors, govern most soil CRT on an individual basis.

The results of this work indicate that while soil CRT are sensitive to spatiotemporal filters, they do not account for observed whole community variability across space and time. This is significant in that it implies two possible ecological roles for soil CRT; either these rare members are not important to overall microbial community dynamics or their functional capacity is unrelated to their abundance. These results indicate that spatiotemporal community turnover may be largely directed by abundant taxa that fluctuate in abundance but do not cross the threshold of rarity, and by unique communities of perennially rare taxa. Future studies of soil CRT would benefit from a more frequently sampled time series to increase statistical power. Analyses of activity in concert with community analyses will also give insight into the relationships between microbial abundance, diversity and function.

## Author Contributions

RK collected and processed the samples. RK and SM designed the experiments, analyzed the data, and were both involved in the writing process.

## Conflict of Interest Statement

The authors declare that this work was funded in part by a grant from Mainland Minerals Ltd., a fertilizer company based in New Zealand. The authors declare that Mainland Minerals Ltd. was not involved in the study design or collection, analysis, or interpretation of the data.
