# Supplementary material for: Conditionally Rare Taxa Contribute but Do Not Account for Changes in Soil Prokaryotic Community Structure
Source: Front Microbiol. 2018 Apr 27;9:809. doi: 10.3389/fmicb.2018.00809 (PMC5934496; doi:10.3389/fmicb.2018.00809)
Supplement: Supplementary file 6 [file Presentation_1.pdf]

Supplementary Information

Conditionally rare taxa contribute but do not account for prokaryotic community changes in soils

Contents:

Figure S1: Workflow for identifying CRT
Figures S2-3: Rank abundance curves for individual sites and whole community
Figure S4-5: ANOSIM test for correlations between time and Bray-Curtis distances
Figure S6: ANOSIM tests for correlations between spatial factors and Bray-Curtis distances
Table S1: Sampling scheme (separate file).
Table S2: Full list of physicochemical factors for all sites (separate file).
Table S3: Mantel correlations between site-level whole communities and site-level whole communities excluding CRT (separate file).
Table S4: Mantel and ANOSIM correlations between whole community/CRT and spatial factors (separate file).
Table S5: Full list of conditionally rare OTUs that are correlated with time (separate file).

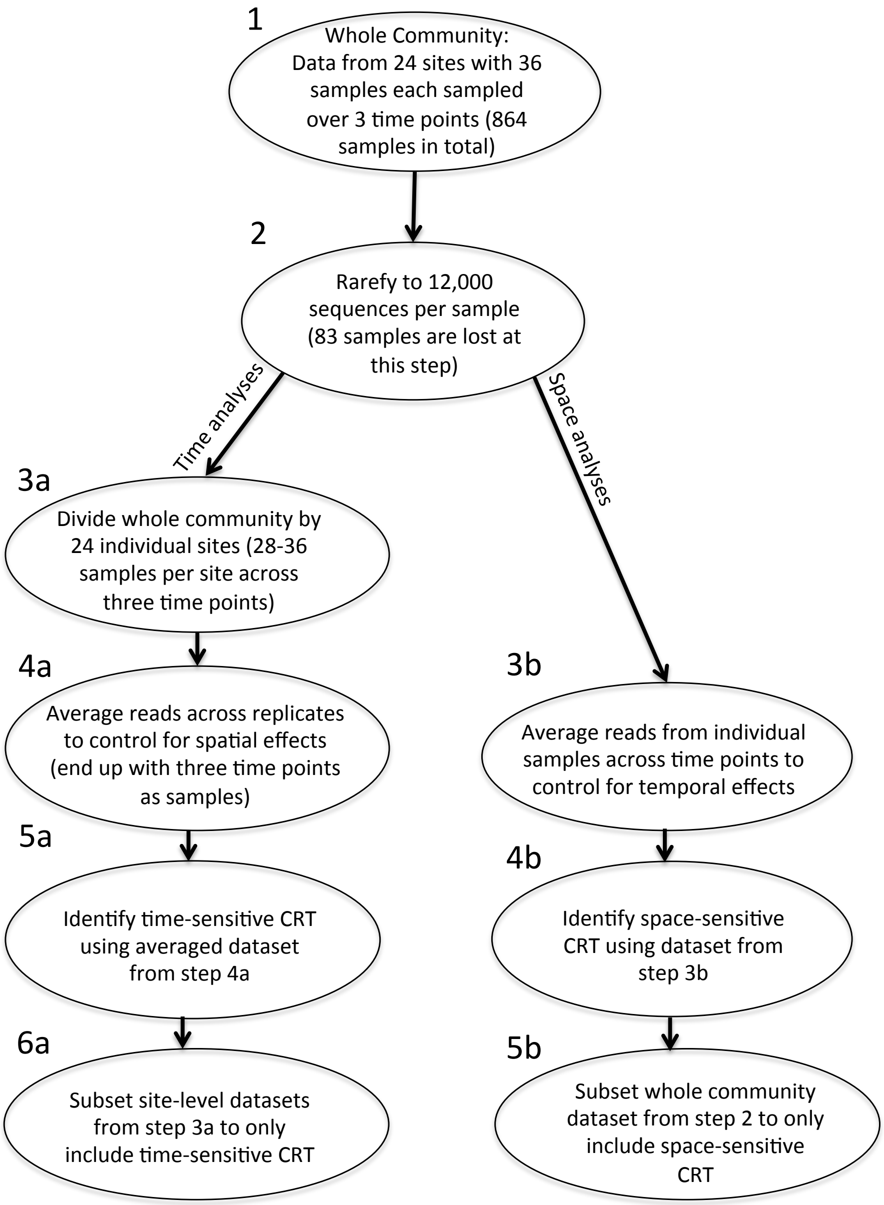

Figure S1: Workflow showing the method for identifying time and space sensitive CRT.

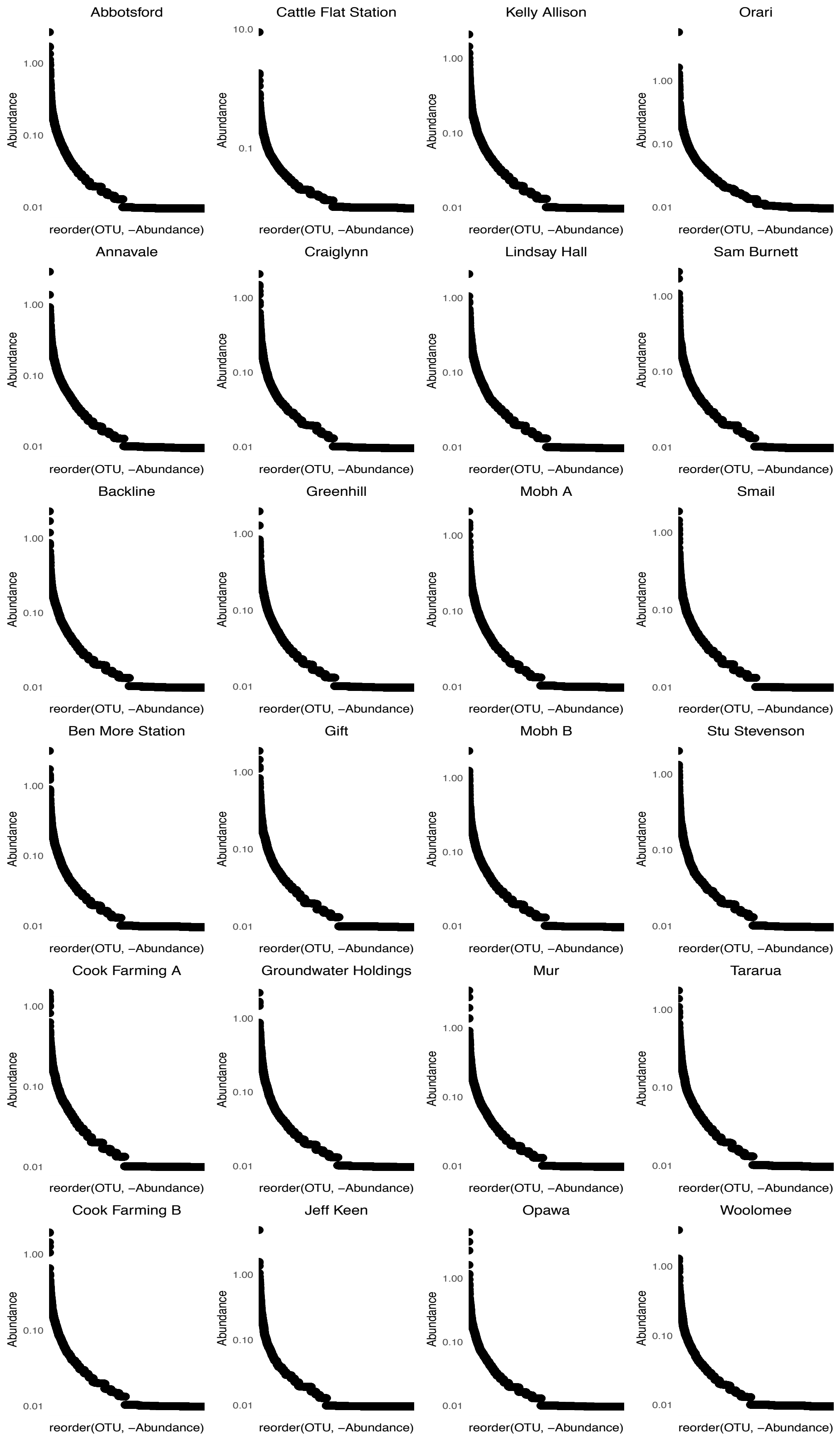

Figure S2: Rank abundance curves for individual sites. Abundance is presented as a percentage.

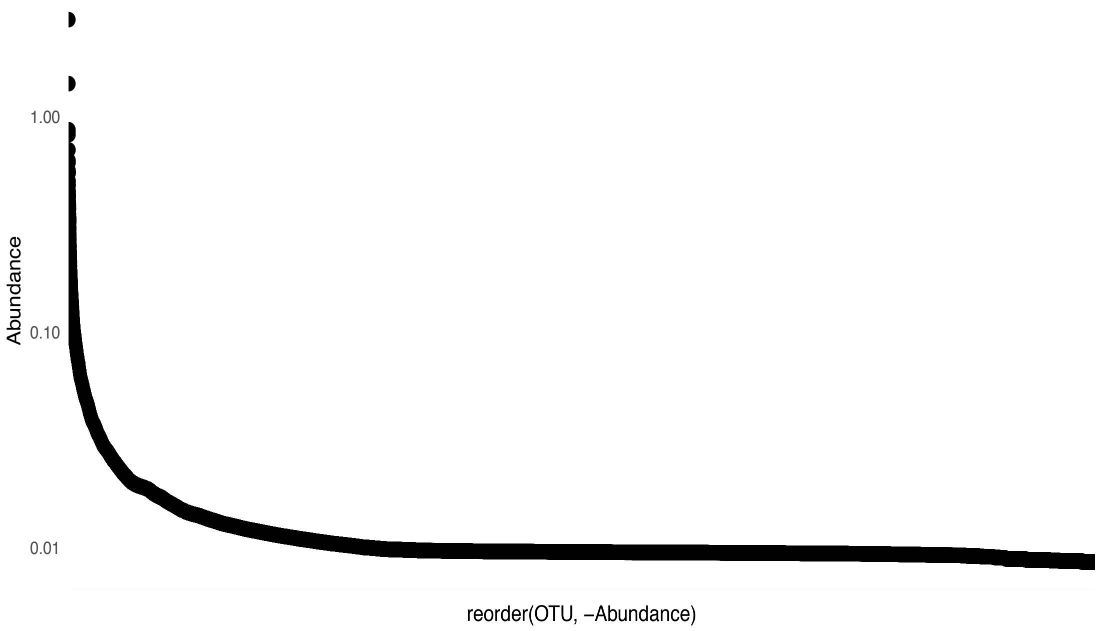

Figure S3: Rank abundance curve for whole community. Abundance is presented as a percentage.

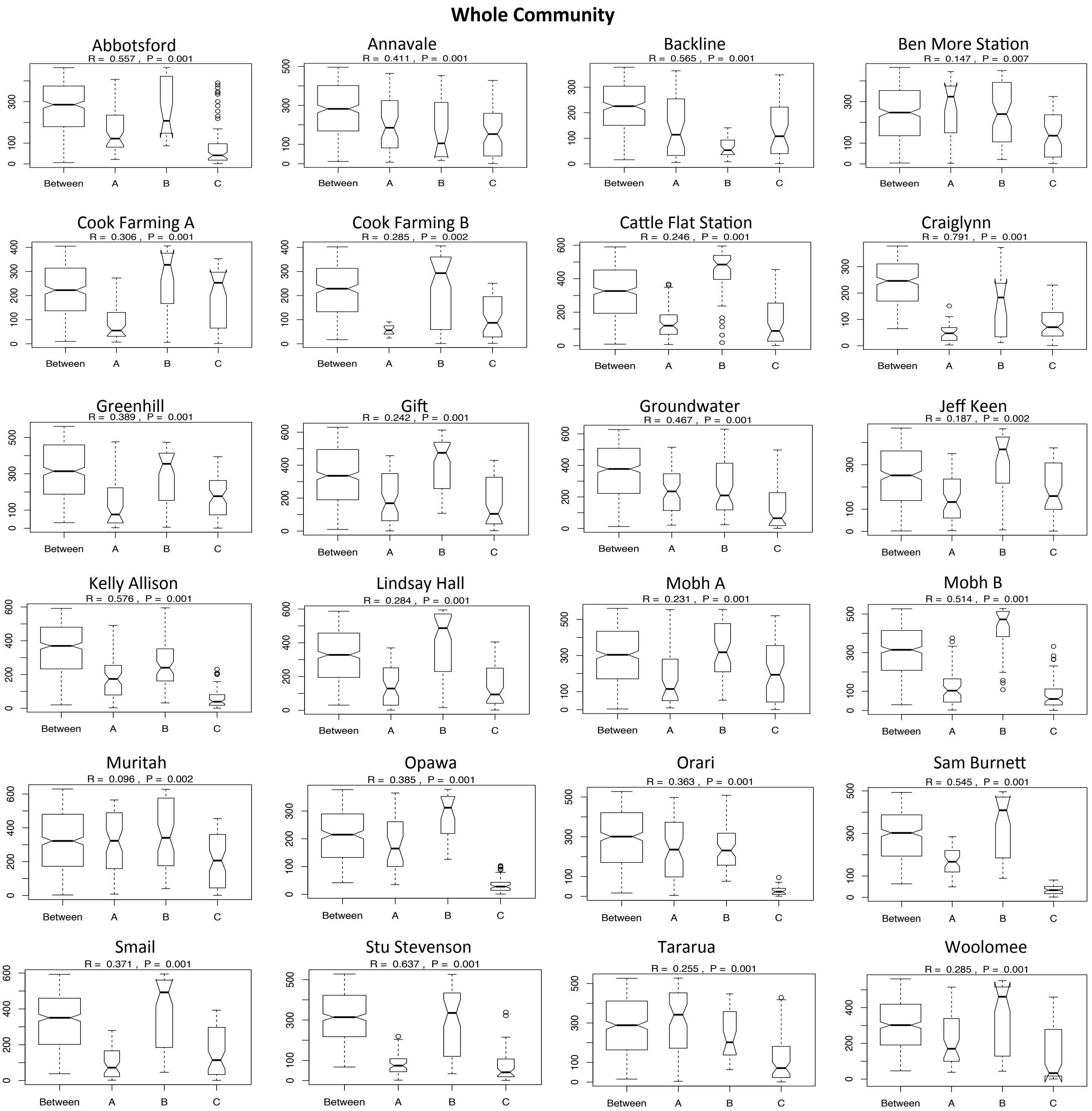

Figure S4: ANOSIM tests for correlations between time point and Bray-Curtis distances for the whole community from each site. Ranked dissimilarities plotted against the three time points: A = May 2014, B = November 2014, C = May 2015. Boxplots are drawn with widths proportional to the square roots of the number of observations in each time points and with 95% confidence intervals.

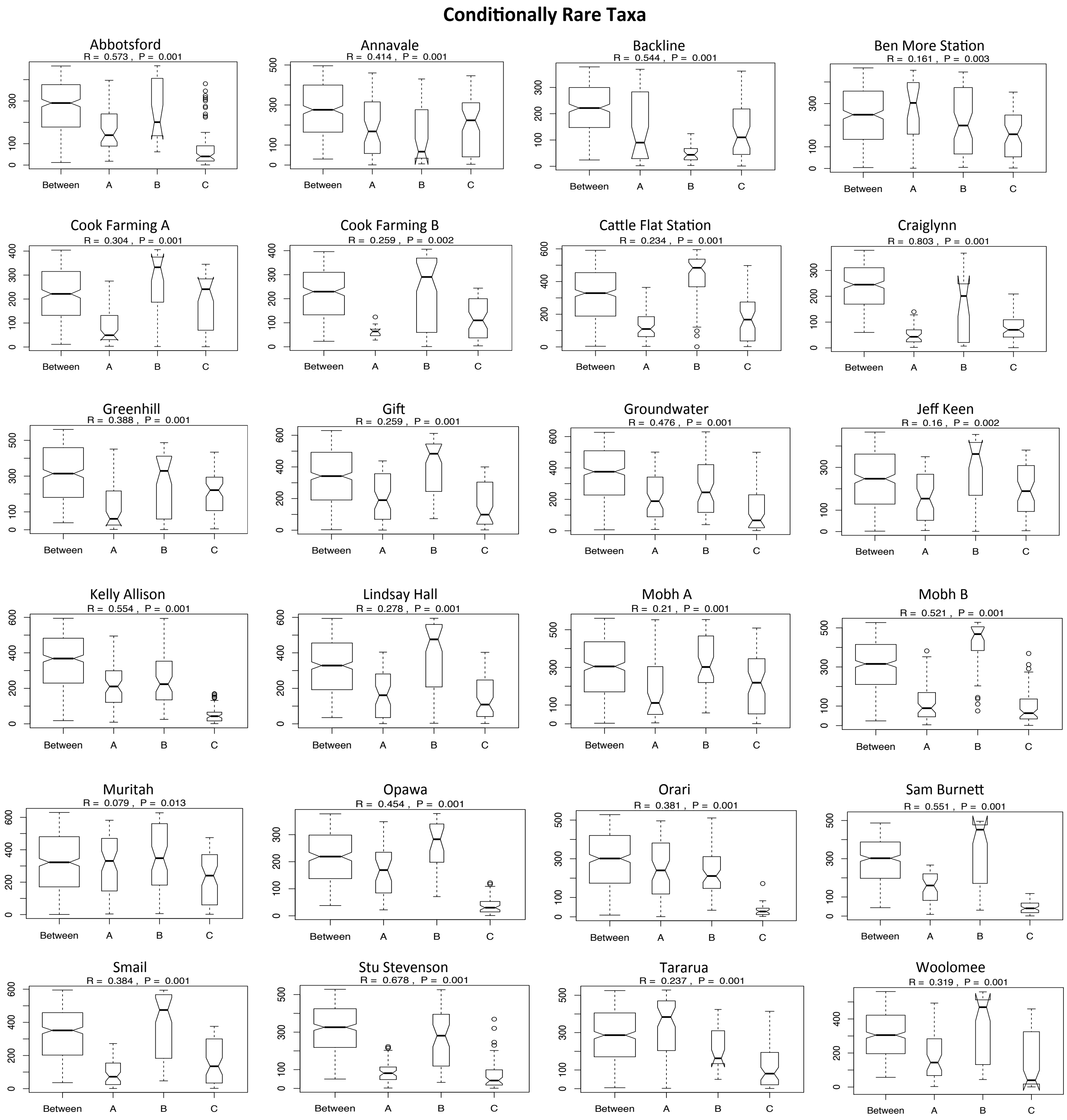

Figure S5: ANOSIM tests for correlations between time point and Bray-Curtis distances for CRT from each site. Ranked dissimilarities plotted against the three time points: A = May 2014, B = November 2014, C = May 2015. Boxplots are drawn with widths proportional to the square roots of the number of observations in each time points and with 95% confidence intervals.

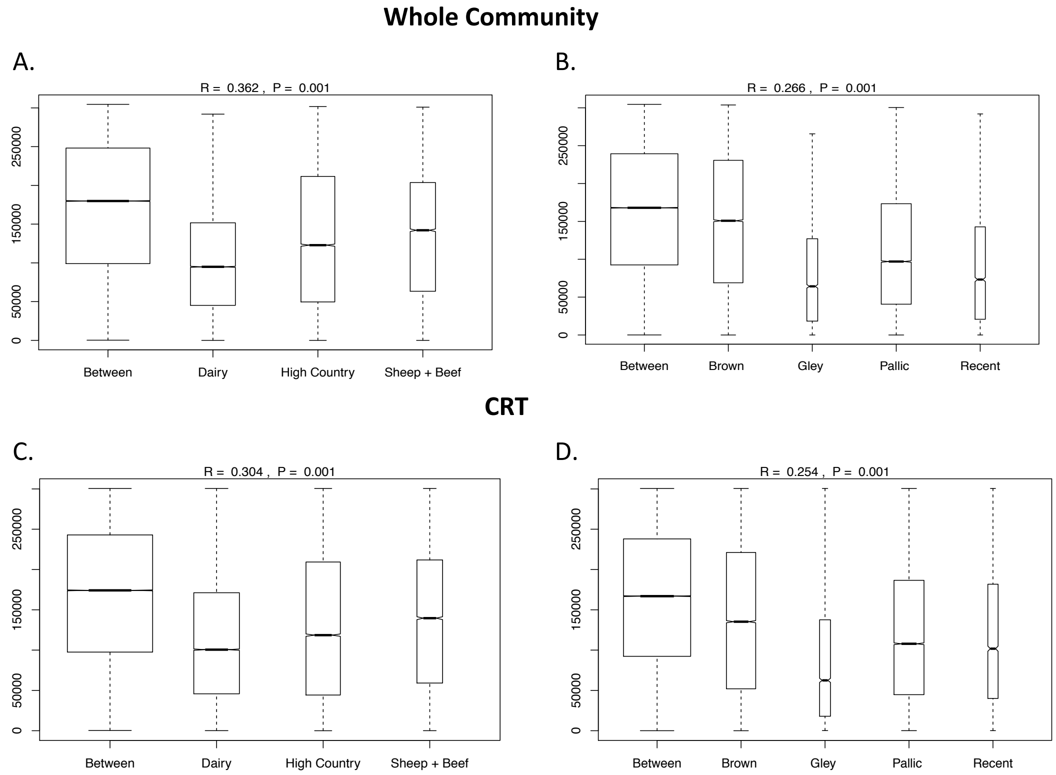

Figure S6: ANOSIM tests for correlations between spatial factors and Bray-Curtis distances for the whole community (A, B) and CRT (C, D). Ranked dissimilarities plotted against the land use and soil order. Boxplots are drawn with widths proportional to the square roots of the number of observations in each time points and with 95% confidence intervals.
